# Duration of exposure to night work and cardiovascular risk factors: results from 52,234 workers of the CONSTANCES study

**DOI:** 10.1186/s12889-025-21511-2

**Published:** 2025-01-28

**Authors:** Eve Bourgkard, Stéphanie Boini, Michel Grzebyk, Mathieu Dziurla, Jean Ferrières, Marcel Goldberg, Céline Ribet, Yolande Esquirol

**Affiliations:** 1 Department of Occupational Epidemiology, French Research and Safety Institute for the Prevention of Occupational Accidents and Diseases (INRS), Vandœuvre-Les-Nancy, France; 2 UMR 1295, Paul Sabatier III University-Inserm, CERPOP: Centre for Epidemiology Research in Population Health, Toulouse, France; 3 Cardiovascular Department, CHU Toulouse, Toulouse, France; 4 Inserm, Université Paris Cité, Université Paris Saclay, Université de Versailles-Saint-Quentin-en-Yvelines (UVSQ), UMS 11 “Population-Based Epidemiological Cohorts Unit”, Villejuif, France; 5 Occupational Health Department, CHU Toulouse, Toulouse, France

**Keywords:** Obesity, Blood pressure, Lipids, Glycaemia, Night-shift work, Occupational exposure

## Abstract

**Background:**

The cardiovascular consequences of night work are increasingly well-known. Implementing effective preventive strategies, however, requires further investigation of the effects of exposure duration. This study sought to assess the cumulative dose–effect of night work exposure on the prevalence of cardiovascular risk factors among current and former night workers in France.

**Methods:**

We used cross-sectional data from the CONSTANCES cohort to design analyses on 52,234 workers exposed or not exposed to night work during their working life. The cumulative duration of night work exposure was assessed among permanent, rotating and former night workers. BMI, blood pressure, lipids, glycaemia and SCORE2 were measured in health screening centres.

**Results:**

Excess risks of moderate-high SCORE2 were observed for permanent (+ 43%), rotating (+ 72%) and former night workers (+ 101%). Among male permanent night workers, excess risks for obesity (+ 76%) and central obesity (64%) were recorded at five years of exposure and for T2DM (+ 119%) at 10 years of exposure. Male rotating night workers showed excess risks at five years of exposure for obesity/central obesity (about + 45%) and high triglyceridaemia (+ 52%). Female former night workers were at excess risk at five years of exposure for obesity/central obesity (about + 45%), HBP (+ 34%) and low-HDL-C (+ 35%).

**Conclusions:**

The effects on cardiovascular risk factors varied according to the types of night work and within sex groups. Some effects were observed after five years of exposure. These results support the need for early and appropriate monitoring of cardiovascular risk factors among current and former night workers.

**Graphical Abstract:**

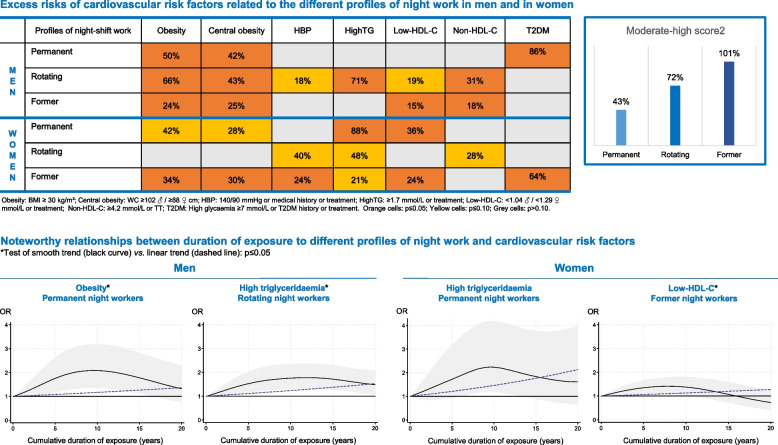

**Supplementary Information:**

The online version contains supplementary material available at 10.1186/s12889-025-21511-2.

## Background

Recent research increasingly provides evidence of a link between shift work and both cardiovascular diseases [1–3] and associated risk factors, notably excess risks of diabetes, hypertension and being overweight or obese [4]. The association between shift work and lipid disorders calls for further exploration [4]. Most of the studies that have been carried out to date have used a broad definition of shift work, with no clear information on whether or not night work was included in the working pattern. Moreover, few studies have explored the cardiovascular impact of the duration of exposure to night work. Indeed, the conclusions of recent meta-analyses based on the same two studies on specific populations for diabetes [5, 6] and on only four studies into obese/overweight subjects provided little clarity concerning the effect of shift work exposure duration [7]. More detailed investigation is required to understand the effects of exposure durations on cardiovascular risk factors (CVRFs), according to both sex and patterns of night work across an individual’s career. Moreover, the persistence of cardiovascular effects among former night workers has been poorly investigated and remains a major concern. Our hypotheses were that (1) permanent and rotating night work would have different effects on CVRFs and these effects would depend on the duration of exposure over an individual’s working life; (2) the effects on CVRFs would persist among former night workers.

This present study aimed to assess the cumulative dose–effect that exposure to different types of night work over a worker’s entire career might have on CVRFs (obesity, central obesity, high blood pressure (HBP), high triglyceridaemia, low high-density lipoprotein-cholesterol (low-HDL-C), non-high-density lipoprotein-cholesterol (Non-HDL-C), type 2 diabetes (T2DM) and the 10-year cardiovascular disease risk assessment (SCORE2)). This study also aimed to assess the association between different types of night work and the prevalence of CVRFs.

## Methods

### Study population and setting

The present study is a cross-sectional study with an assessment of exposure to night work over the working life. It was based on data from the population-based CONSTANCES cohort, which comprises more than 220,000 French adults, randomly selected from the French national inter-scheme registry of health insurance beneficiaries [8]. The present study included participants who were in active employment at the time of their recruitment (2012–2018) and who had worked nights or only days during their working life. Of a total of 62,429 participants (eFigure 1), those with a history of personal cardiovascular diseases (*n* = 727), type 1 diabetes (*n* = 118) or with missing values for CVRFs (*n* = 4,236) or main adjustment factors (*n* = 5,114) were excluded. The final study sample consisted of 52,234 subjects: 24,892 men (47.7%) and 27,342 women (52.3%).

### Data collection

At baseline, participants completed self-administered questionnaires regarding socio-demographic factors, lifestyle behaviours and lifelong occupational history and working patterns [8]. They were also comprehensively examined at health screening centres to determine: (i) personal medical histories; (ii) clinical parameters (weight, height and waist circumference using standard equipment, and resting systolic and diastolic arterial blood pressure using an automatic oscillometric blood pressure monitor); and (iii) biomarkers (fasting lipid and glucose parameters assessed by blood sample) using standardised methods described elsewhere [9].

### Outcomes

The CVRFs studied were defined as dichotomous variables: obesity (body mass index (BMI) ≥ 30 kg/m^2^); central obesity (waist circumference ≥ 102 cm for men and ≥ 88 cm for women); high arterial blood pressure (HBP) defined as systolic blood pressure (SBP) ≥ 140 mm Hg or diastolic blood pressure (DBP) ≥ 90 mm Hg or arterial hypertension history or treatment; high triglyceridaemia (≥ 1.7 mmol/L or treatment); low high-density lipoprotein cholesterol (low-HDL-C < 1.04 mmol/L for men and < 1.29 mmol/L for women or treatment); non-high-density lipoprotein cholesterol (Non-HDL-C ≥ 4.2 mmol/L or treatment); T2DM (≥ 7 mmol/L or type 2 diabetes history or treatment). The 10-year risk of cardiovascular disease was assessed using the SCORE2, following recently established guidelines [10, 11].

### Night work exposure

The participants were asked two questions with yes/no answers: (1) “Do you have (or have you had) a work schedule that often requires (or required) you to stay up at night at least 50 days each year?”; and (2) “Do you have (or have you had) alternating shift work (by teams, brigades, shifts)?” If a participant responded “Yes” to either question, they were invited to provide details of the corresponding periods over their occupational career [8]. Based on the responses to these two questions, participants were classified into four groups: (i) permanent night-working schedule (labelled “permanent night work”), i.e., “Yes” to question 1 and “No” to question 2 for the current job at baseline; (ii) shift work alternating nights and days (“rotating night work”), i.e., “Yes” to questions 1 and 2 for the current job at baseline; (iii) day work but with a history of permanent night or rotating night work ( “former night work”), i.e., “No” to questions 1 and 2 for the current job at baseline and “Yes” to question 1 or “Yes” to question 1 and 2 for former jobs; (iv) day work without night work over the entire career (“day work”), i.e. “No” to questions 1 and 2 for current and former jobs. These latter participants represented the reference group.

Moreover, the cumulative duration of exposure to night work reported in the periods was calculated over the individual’s working life.

Given the potentially varied experiences of night work – either permanent or rotating – over a single subject’s working life, the homogeneity of each night exposure group was assessed. To do so, the ratio of the cumulative duration of permanent night work to the cumulative duration of permanent and rotating night work was calculated and expressed as a percentage. This method was also used to calculate the ratio for rotating night work.

### Adjustment factors

Cardiovascular risk confounders were used as adjustment factors. Individual characteristics, such as age and educational level (until high school degree / Bachelor’s degree / Master’s university degree and above), were considered. Lifestyle behaviours were assessed by smoking status (non-smokers / current and former smokers), regular leisure time physical activity (yes / no) and alcohol consumption habits (abstinence / no abusive consumption / risk of abusive consumption) based on AUDIT-C [12]. Menopausal status and substitutive oral hormonal treatment (yes / no) were also considered for women.

### Statistical analyses

Participants’ characteristics were described using percentages for categorical variables and mean and range for continuous variables.

#### Association between types of night work and CVRFs

Multiple logistic regression models were performed to assess the association between each CVRF and the different types of night work compared to day work (reference group). ORs and 95%CIs were initially estimated in a partial model that took into account individual factors, before employing a full model that took into account individual and lifestyle behavioural factors as well as menopausal status and the use of substitutive oral hormonal treatment. The probability of a moderate-high vs. low SCORE2 according to the different types of night work was assessed using logistic regression.

#### Cumulative duration of exposure to night work during working life and CVRFs

Generalised additive models (GAM) [13] were used to assess the contribution of the cumulative duration of exposure to night work on each CVRF by types of night work: permanent night work; rotating night work; and former night work. Thus, the cumulative duration of night work was included in logistic regression models as smooth non-linear functions. Models were adjusted for age (also considered as a smooth non-linear function), education level, smoking status, regular leisure time physical activity, alcohol consumption habits, menopausal status and substitutive oral hormonal treatment, except for SCORE2. The results were represented graphically with the cumulative duration of night exposure on the x-axis and ORs on the y-axis. In supplementary tables, point estimated ORs and 95%CIs at five, 10, 15 and 20 cumulative years of exposure duration as well as the linear trend per year were provided. The smooth model’s gain over the linear trend was also tested.

All statistical analyses were performed separately for men and women, with the exception of SCORE2. The statistical significance was set at 5% for the two-tailed *p*-value. All analyses were conducted using Stata version 18 (Stata Corporation, College Station, TX, USA).

### Ethical approval

CONSTANCES has obtained authorisation from the French National Data Protection Authority (n°910486) for research on human subjects and was approved by the National Council for Statistical Information, the National Medical Council and the Institutional Review Board of the National Institute for Medical Research (INSERM). All participants gave their written consent.

## Results

The characteristics of male workers according to the different types of night work are reported in eTable 1. The male study sample comprised 21,957 (88.2%) day workers, 415 (1.7%) permanent night workers, 615 (2.5%) rotating night workers and 1,905 (7.6%) former night workers. In the permanent night group, on average 95.2% of night work duration was carried out in a permanent night work pattern. In the rotating night group, 97.5% of night work duration was carried out in a rotating night work pattern.

The characteristics of female workers according to the different types of night work are reported in eTable 2. The female study sample comprised 25,705 (94.0%) day workers, 270 (1.0%) permanent night workers, 300 (1.1%) rotating night workers and 1,067 (3.9%) former night workers. In the permanent night group, on average 97% of night work duration was carried out in a permanent night work pattern. In the rotating night group, 98.5% of night work duration was carried out in a rotating night work pattern.

The mean and standard deviation of each clinical and biological CVRF are given in supplementary eTable 3. The frequency of each clinical and biological CVRF was statistically significantly different across the categories of working schedules for men (eTable 4) and women (eTable 5). The comparison between included and excluded participants is summarised in eTable 6.

### Association between different types of night work and CVRFs

Table 1 presents the ORs and 95%CIs obtained by partial and full models to evaluate the associations between the different types of night work and the main CVRFs among male and female workers. The results of the full models including the effects of adjustment factors are presented in eTable 7.

**Table 1:** Association between different types of night work and cardiovascular risk factors among male and female workers (reference: day work)

		**Men (*n* = 24,892)**		**Women (*n* = 27,342)**	
		**Partial model**^**a**^	**Full model**^**b**^	**Partial model**^**a**^	**Full model**^**b**^
		**OR (95%CI)**	**OR (95%CI)**	**OR (95%CI)**	**OR (95%CI)**
**BMI ≥ 30 kg/m**^**2**^	Day work	1	1	1	1
	Permanent night	1.54 (1.17–2.03)^**^	1.50 (1.14–1.97)^**^	1.41 (0.99–2.01)^*^	1.42 (0.98–2.03)^*^
	Rotating night	1.64 (1.32–2.05)^**^	1.66 (1.32–2.07)^**^	1.18 (0.82–1.69)	1.20 (0.84–1.73)
	Former night	1.26 (1.10–1.45)^**^	1.24 (1.08–1.42)^**^	1.33 (1.10–1.59)^**^	1.34 (1.11–1.61)^**^
**Waist circumference ≥ 102 ♂ / 88 ♀ cm**	Day work	1	1	1	1
	Permanent night	1.46 (1.12–1.92)^**^	1.42 (1.08–1.87)^**^	1.28 (0.95–1.71)^*^	1.28 (0.95–1.72)^*^
	Rotating night	1.43 (1.14–1.79)^**^	1.43 (1.14–1.80)^**^	1.13 (0.84–1.52)	1.14 (0.85–1.54)
	Former night	1.27 (1.12–1.45)^**^	1.25 (1.10–1.43)^**^	1.30 (1.13–1.50)^**^	1.30 (1.12–1.51)^**^
**High arterial blood pressure (SBP / DBP ≥ 140 / 90 mm Hg or hypertension history or treatment)**	Day work	1	1	1	1
	Permanent night	0.89 (0.71–1.11)	0.89 (0.71–1.11)	1.24 (0.87–1.75)	1.24 (0.87–1.76)
	Rotating night	1.16 (0.97–1.39)^*^	1.18 (0.98–1.41)^*^	1.39 (0.98–1.96)^*^	1.40 (0.99–1.98)^*^
	Former night	0.97 (0.88–1.08)	0.98 (0.88–1.09)	1.23 (1.05–1.44)^**^	1.24 (1.06–1.45)^**^
**High triglyceridaemia (≥ 1.7 mmol/L or treatment)**	Day work	1	1	1	1
	Permanent night	1.14 (0.89–1.45)	1.11 (0.86–1.41)	1.88 (1.28–2.77)^**^	1.88 (1.28–2.77)^**^
	Rotating night	1.71 (1.42–2.05)^**^	1.71 (1.42–2.05)^**^	1.46 (0.96–2.22)^*^	1.48 (0.98–2.25)^*^
	Former night	1.12 (0.99–1.26)^*^	1.10 (0.98–1.24)	1.21 (0.97–1.51)^*^	1.21 (0.97–1.51)^*^
**Low-HDL-C (< 1.04 ♂ / 1.29 ♀ mmol/L or treatment)**	Day work	1	1	1	1
	Permanent night	1.06 (0.83–1.36)	1.02 (0.80–1.31)	1.36 (1.02–1.80)^**^	1.36 (1.03–1.81)^**^
	Rotating night	1.21 (0.99–1.48)^*^	1.19 (0.98–1.45)^*^	1.03 (0.77–1.37)	1.02 (0.77–1.36)
	Former night	1.18 (1.05–1.32)^**^	1.15 (1.02–1.29)^**^	1.26 (1.08–1.46)^**^	1.24 (1.07–1.45)^**^
**Non-HDL-C (≥ 4.2 mmol/L or treatment)**	Day work	1	1	1	1
	Permanent night	1.03 (0.84–1.27)	1.02 (0.83–1.25)	0.94 (0.70–1.26)	0.94 (0.70–1.27)
	Rotating night	1.32 (1.11–1.56)^**^	1.31 (1.11–1.55)^**^	1.28 (0.97–1.69)^*^	1.28 (0.97–1.69)^*^
	Former night	1.19 (1.08–1.32)^**^	1.18 (1.07–1.30)^**^	0.99 (0.86–1.14)	1.00 (0.87–1.15)
**Type 2 diabetes (glycaemia ≥ 7 mmol/L or type 2 diabetes history or treatment)**	Day work	1	1	1	1
	Permanent night	1.95 (1.22–3.12)^**^	1.86 (1.16–2.98)^**^	1.61 (0.71–3.65)	1.63 (0.71–3.70)
	Rotating night	1.28 (0.80–2.04)	1.27 (0.80–2.03)	1.06 (0.39–2.86)	1.09 (0.40–2.97)
	Former night	1.15 (0.90–1.47)	1.13 (0.88–1.44)	1.62 (1.11–2.36)^**^	1.64 (1.12–2.40)^**^

*BMI* body mass index, *SBP* systolic blood pressure, *DBP* diastolic blood pressure, *HDL* high-density lipoprotein

^**^p ≤ 0.05

^*^p < 0.10

^a^partially adjusted logistic model: age, educational level

^b^fully adjusted logistic model: age, educational level, smoking status, regular leisure time physical activity and alcohol consumption habits, as well as menopausal status and substitutive oral hormonal treatment in female models

Permanent night workers presented increased risks of having a BMI ≥ 30 kg/m^2^ and a waist circumference ≥ 102/88 cm of 50% and 42% for men (*p* ≤ 0.05) and 42% and 28% (*p* ≤ 0.10) for women, respectively. Men showed a significant risk of T2DM (1.86[1.16–2.98]) while, for women, the risks of high triglyceridaemia and low-HDL-C were estimated at 1.88[1.28–2.77] and 1.36[1.03–1.81], respectively (Table 1). For all participants, the risk of a moderate-high SCORE2 was estimated at 1.43[1.23–1.66] (eTable 8).

Among the male rotating night workers, the main significant associations were found for BMI ≥ 30 kg/m^2^ (1.66[1.32–2.07]), waist circumference ≥ 102 cm (1.43[1.14–1.80]), high triglyceridaemia (1.71[1.42–2.05]) and Non-HDL-C (1.31[1.11–1.55]); among women, however, increased risks with *p* ≤ 0.10 were observed for HBP (+ 40%), high triglyceridaemia (+ 48%) and Non-HDL-C (+ 28%) (Table 1). For all participants, the risk of a moderate-high SCORE2 was estimated at 1.72[1.51–1.97] (eTable 8).

Among former night workers, significant excess risks were observed for BMI ≥ 30 kg/m^2^ (+ 24% in men, + 34% in women), waist circumference ≥ 102/88 cm (+ 25% in men, + 30% in women), high arterial blood pressure (+ 24% in women), low-HDL-C (+ 15% in men, + 24% in women), Non-HDL-C (+ 18% in men) and T2DM (+ 64% in women). Women presented a 21% excess risk for high triglyceridaemia (*p* < 0.10) (Table 1). For all participants, the risk of a moderate-high SCORE2 was estimated at 2.01[1.87–2.18] (eTable 8).

### Cumulative duration of exposure to night work and each CVRF

#### Among permanent night workers

**Fig. 1:**
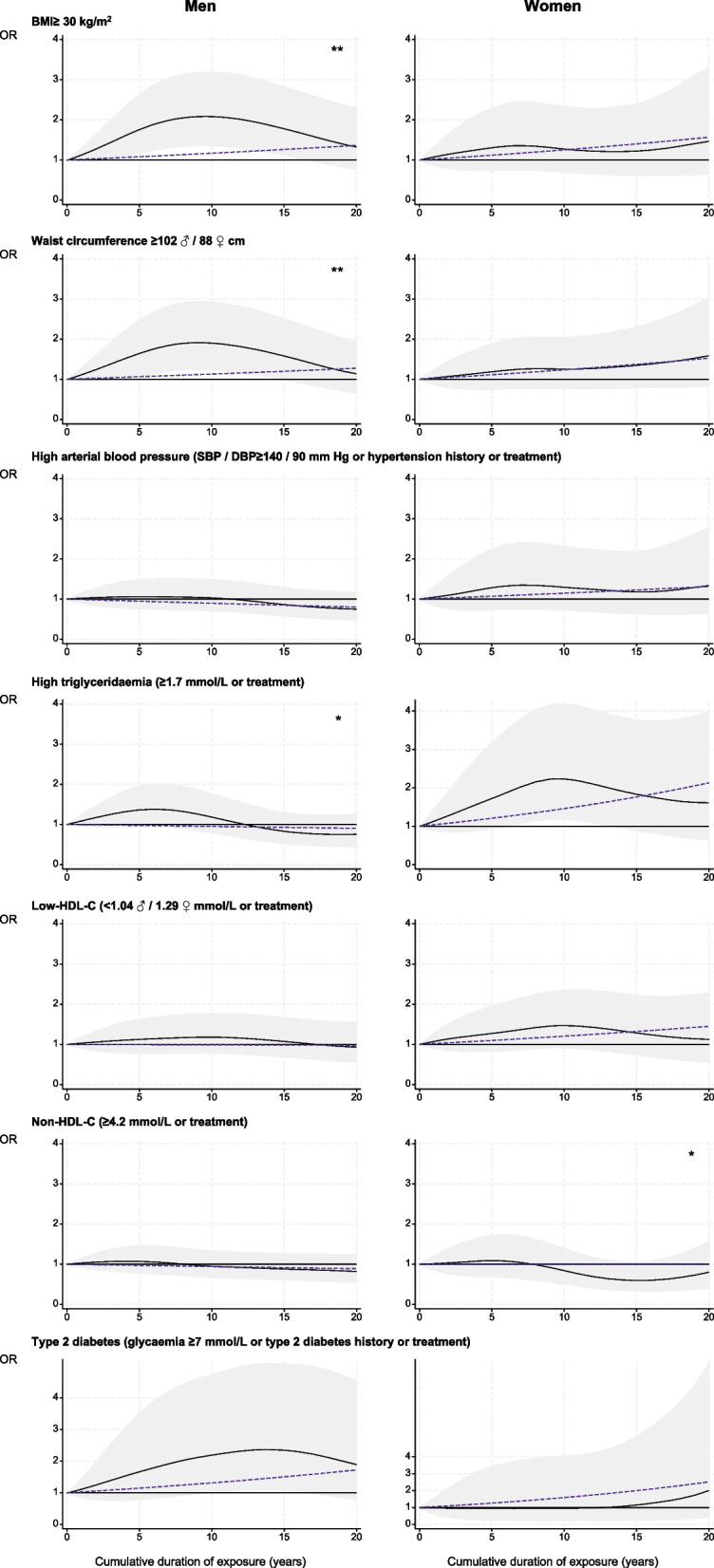
Cumulative duration of night work exposure and cardiovascular risk factors among permanent night workers, estimated using generalised additive models. BMI: body mass index; SBP: systolic blood pressure; DBP: diastolic blood pressure; HDL: high-density lipoprotein. Black curve: OR of each cardiovascular risk factor for one given cumulative duration compared to no exposure under smooth effect hypothesis. Shadow: corresponding 95% confidence interval. Dashed line: OR of each cardiovascular risk factor for one given cumulative duration compared to no exposure under linear effect hypothesis. Test of smooth trend (black curve) *vs.* linear trend (dashed line): ** *p* ≤ 0.05; * *p* ≤ 0.10

Figure 1 and eTable 9 display the results obtained when assessing associations between the cumulative duration of night work exposure and each CVRF in the fully adjusted model.

For men, the regression spline curves indicate that the relationships between BMI ≥ 30 kg/m^2^ and waist circumference ≥ 102 cm and the exposure duration were non-linear (gain models vs. linear trends: *p* ≤ 0.05, eTable 9). The risk of both cardiovascular factors increased early on, becoming significant at approximately five years of exposure and peaking at 10 years. The risk for T2DM was significant after 10 years of exposure, reaching its peak at 15 years (Fig. 1). Finally, an elevated risk of high triglyceridaemia was noticed at five years of exposure (1.36[0.95–1.95]) (eTable 9).

For women, the risk of high triglyceridaemia increased by 4% per year, peaking at around 10 years of exposure to night work (2.23[1.19–4.18]) (Fig. 1, eTable 9). A linear increase of risks by 2% per year of the duration of night work was observed for BMI ≥ 30 kg/m^2^ (*p* ≤ 0.10), waist circumference ≥ 88 cm (*p* ≤ 0.05) and low-HDL-C (*p* ≤ 0.10), and at 4% for T2DM (*p* ≤ 0.05) (eTable 9).

**Fig. 2:**
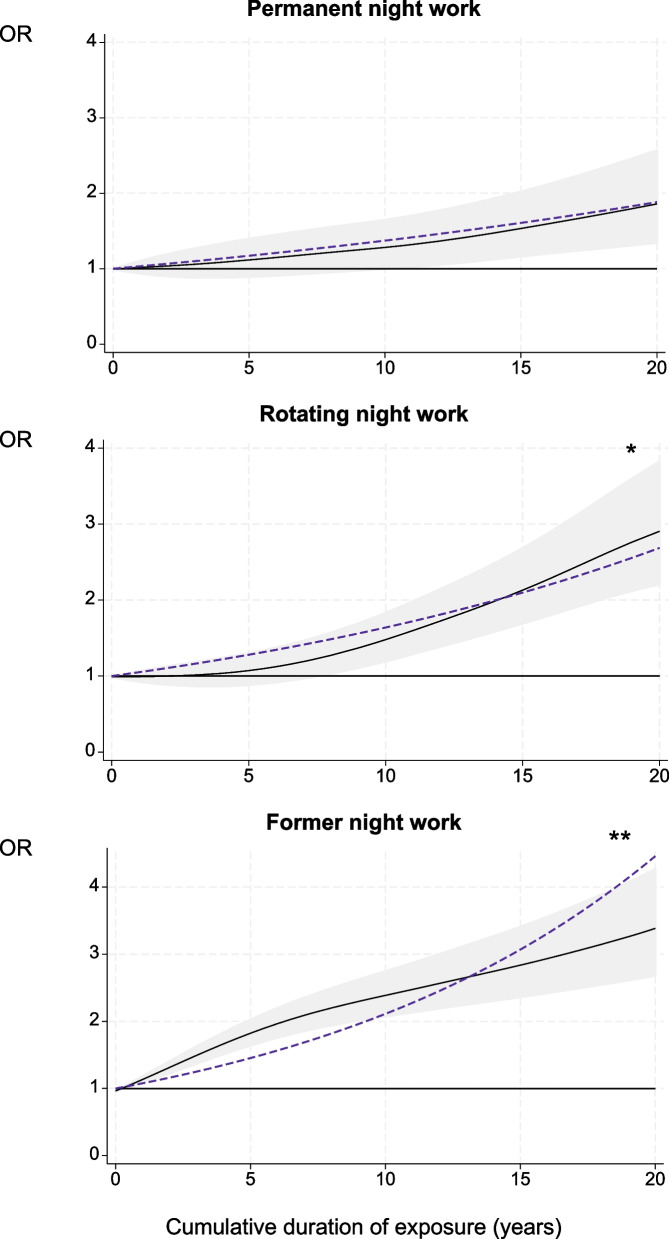
Cumulative duration of night work exposure and 10-year risk of cardiovascular disease (SCORE2), estimated using generalised additive models. Black curve: OR of moderate-high SCORE2 for one given cumulative duration compared to no exposure under smooth effect hypothesis. Shadow: corresponding 95% confidence interval. Dashed line: OR of moderate-high SCORE2 for one given cumulative duration compared to no exposure under linear effect hypothesis. Test of smooth trend (black curve) *vs.* linear trend (dashed line): ** *p* ≤ 0.05; * *p* ≤ 0.10

For all participants, a higher significant excess risk of a moderate-high SCORE2 was observed from 15 years of night work onwards (+ 53%) with a 3% linear increase per year (Fig. 2, eTable 8).

#### Among rotating night workers

**Fig. 3:**
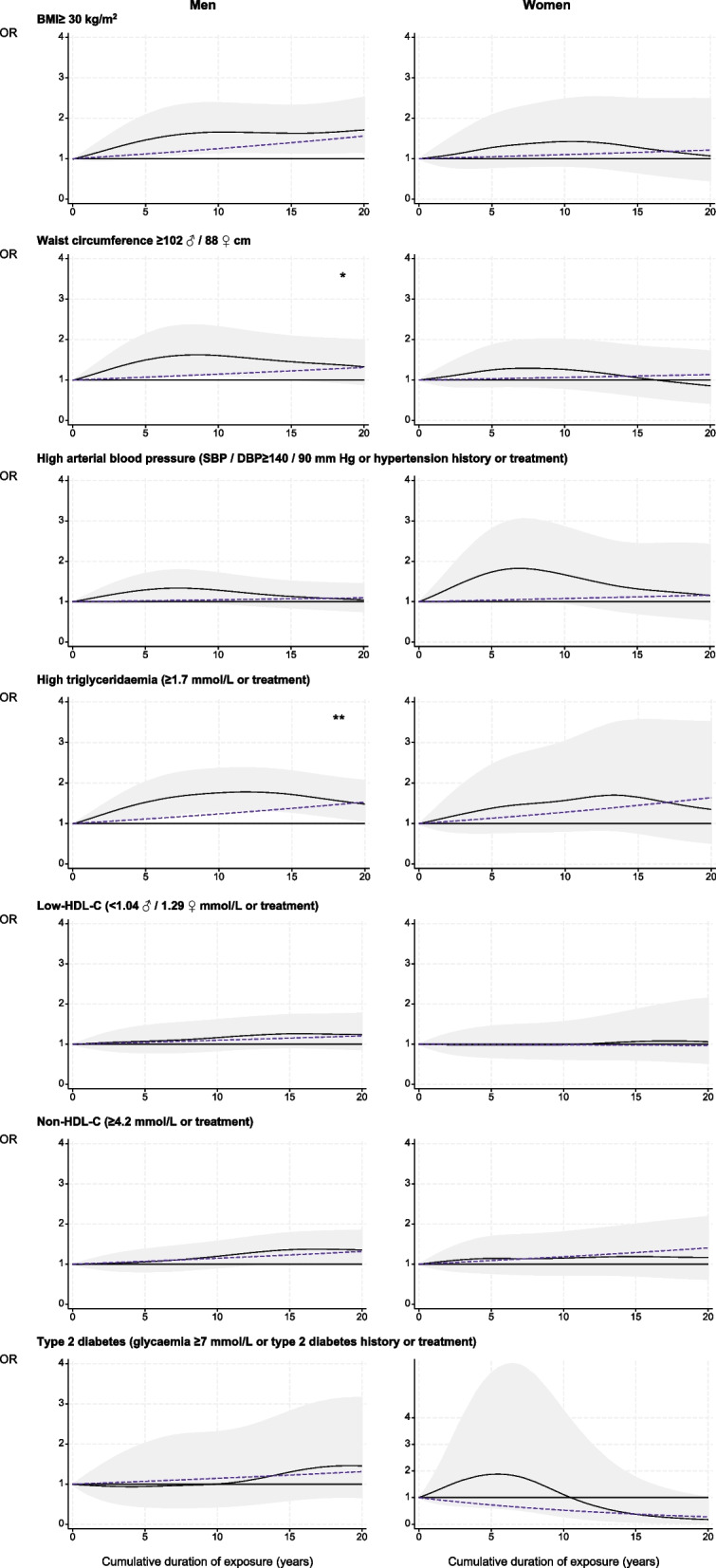
Cumulative duration of night work exposure and cardiovascular risk factors among rotating night workers, estimated using generalised additive models. Black curve: OR of each cardiovascular risk factor for one given cumulative duration compared to no exposure under smooth effect hypothesis. Shadow: corresponding 95% confidence interval. Dashed line: OR of each cardiovascular risk factor for one given cumulative duration compared to no exposure under linear effect hypothesis. Test of smooth trend (black curve) *vs.* linear trend (dashed line): ** *p* ≤ 0.05; * *p* ≤ 0.10

For men, the risk of BMI ≥ 30 kg/m^2^ increased by 2% per year of night work (eTable 10). A significant excess risk (around 45%) was observed starting from five years of night work onwards. The risk of waist circumference ≥ 102 cm increased by 1% per year of night working. Higher significant excess risks were observed for five, 10 and 15 years (49%, 60% and 45%, respectively) (eTable 10). The regression spline curve indicated that the relationship between high triglyceridaemia and the exposure duration was non-linear (gain models vs. linear trends: *p* ≤ 0.05, eTable 10), with a significant excess risk following five years of night work. For Non-HDL-C, a significant excess risk was observed at 15 years (+ 36%) with the risk increasing by 1% each year (Fig. 3).

For women, a significantly increased risk of HBP was observed at five years (+ 73%). When considering *p* ≤ 0.10, the risks of high triglyceridaemia and Non-HDL-C increased by 2% for each year of night work (Fig. 3, eTable 10).

All participants had a higher significant excess risk of a moderate-high SCORE2 from 10 years of night work onwards (+ 48% at 10 years, + 113% at 15 years, + 190% at 20 years) (Fig. 2, eTable 8).

#### Among former workers

**Fig. 4:**
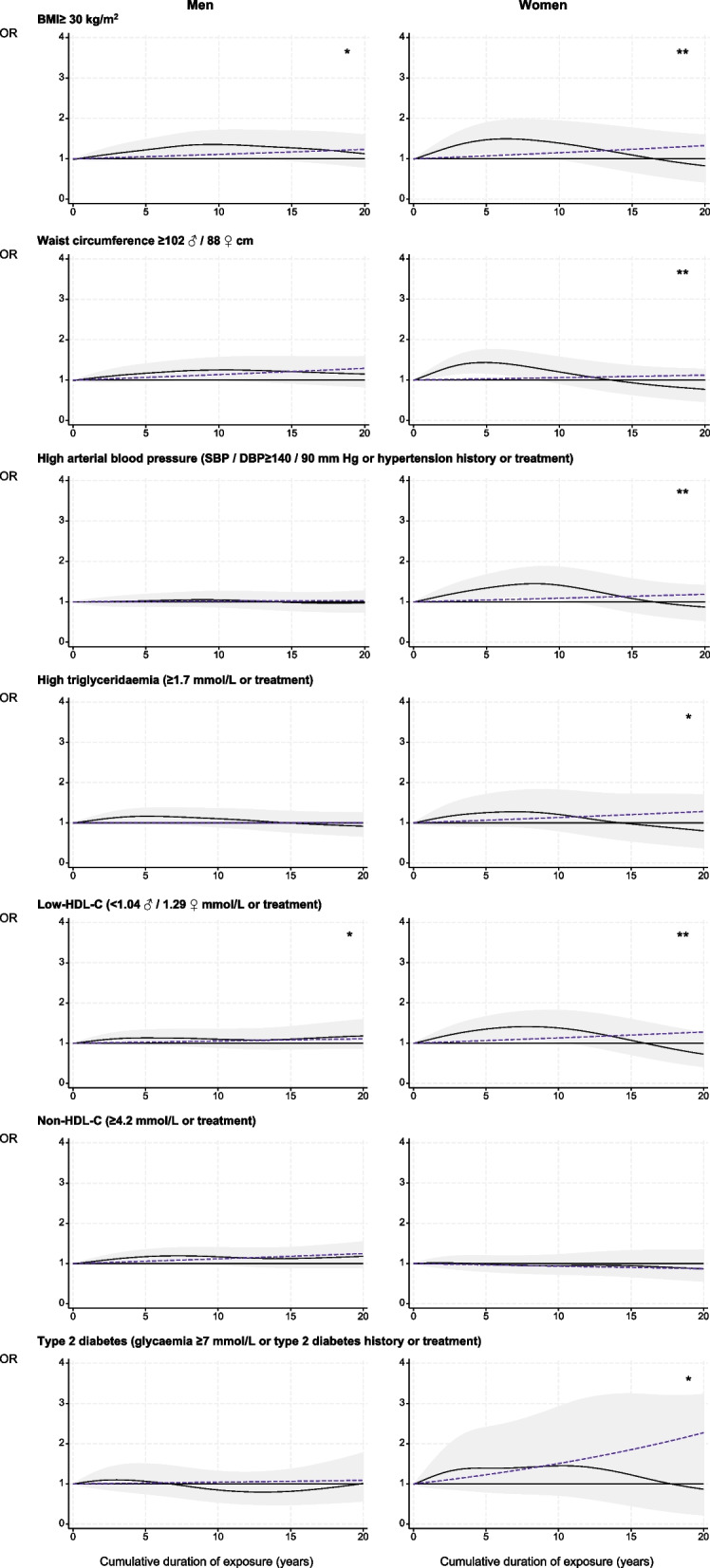
Cumulative duration of night work exposure and cardiovascular risk factors among former night workers, estimated using generalised additive models. Black curve: OR of each cardiovascular risk factor for one given cumulative duration compared to no exposure under smooth effect hypothesis. Shadow: corresponding 95% confidence interval. Dashed line: OR of each cardiovascular risk factor for one given cumulative duration compared to no exposure under linear effect hypothesis. Test of smooth trend (black curve) *vs.* linear trend (dashed line): ** *p* ≤ 0.05; * *p* ≤ 0.10

For men, BMI ≥ 30 kg/m^2^ (+ 22%) and Non-HDL-C (+ 17%) were seen to increase slightly as the duration of exposure to night working rose, and there was a significant excess of risk at five years and 10 years for waist circumference ≥ 102 cm (+ 25%) (Fig. 4, eTable 11). For women, five years of night working significantly increased the risks of BMI ≥ 30 kg/m^2^ (+ 47%), waist circumference ≥ 88 cm (+ 43%), HBP (+ 34%) and low-HDL-C (+ 35%), along with an increased risk of T2DM of 3% for each year of exposure (Fig. 4, eTable 11).

For all participants, higher significant excess risks of a moderate-high SCORE2 were observed from five years of night work onwards (+ 82% at five years, + 139% at 10 years, + 184% at 15 years, + 238% at 20 years) (Fig. 2, eTable 8).

## Discussion

This study set out to assess the relationships between different types of exposure to night working and their cumulative dose–effect, as well as the prevalence of CVRFs (BMI, central obesity, HBP, lipid disorders, T2DM) among current and former night workers (permanent or rotating night work) in France. The observed excess risks of CVRFs were different 1) between men and women, 2) between the different types of night work and 3) according to the exposure duration, with the effects of exposure taking root early on (most often following five years of exposure). Moreover, only few of the effects of exposure duration followed a linear relationship with the CVRFs, and persistent excess risks were witnessed among former night workers. To our knowledge, our study is the first to explore how the duration of exposure to different types of night work throughout the working life affects cardiovascular health. Our results for each cardiovascular risk factor were subsequently compared with the literature by referring to the results of a recent umbrella review published on this topic [4].

Permanent, rotating and former night workers of both sexes were at high risk of obesity (ranging from + 24% to + 66%) and central obesity (ranging from + 25% to + 43%). Three recent meta-analyses reported that excess risks relating to obesity varied from + 12% to + 25% for unspecified shift work [7, 14, 15]. Only one meta-analysis reported results regarding obesity according to different types of night-shift work: + 5% for night shift, based on seven studies; and + 18% for rotating shift with or without night work, based on 17 studies [14]. Our research provides additional information by highlighting the likely high excess risk of obesity in different types of night working. Considering exposure durations was essential to assessing this risk. Indeed, our results demonstrated that the risks of obesity and central obesity increased early on during the first five years of exposure for female former night workers, peaking at 10 years of exposure for male permanent, rotating and former night workers. The increase in risk settled at 1–2% per year of exposure for female permanent night workers and male rotating night workers. According to Sun’s meta-analysis [7], only one study quantified an increase of BMI at 0.24 kg/m^2^ per year of night-shift work [16]. More recently, a study of 529 nurses with over 10 years’ experience of night-shift work demonstrated an increased risk of central obesity (1.76[1.14–2.74]) [17]. A number of authors have highlighted the importance of considering the number of nights worked per month with a higher excess risk when a person works more than eight nights per month [18]. This latter point requires further investigation.

For HBP, excess risks were mainly observed among male and female rotating night workers (+ 18% and + 40%, respectively). We found five-year excess risks of around + 30% among male subjects and around + 70% among female subjects, while we witnessed no excess risk among both male and female permanent night workers. Our findings accord with those provided in a recent umbrella review [4]. Indeed, an excess risk of almost 30% for both sexes was reported for rotating shift work with or without nights [19], but six studies found that permanent night work was associated with no excess risk [20].

For lipid disorders, the excess risks were compared according to sex and type of night work undertaken. Indeed, female permanent night workers had an excess risk of high triglyceridaemia and low-HDL-C, while their male counterparts did not. Among rotating night workers, excess risks were observed for the three lipid biomarkers in men and for two lipid biomarkers (high triglyceridaemia and Non-HDL-C) in women. These excess risks remained for both sexes among former night workers. The effects of night-shift work on lipid disorders have been discussed in the literature [4, 21–24]. Our results make a vital contribution to this debate, however, by affirming that the duration of exposure needs to be considered when studying the impacts of night work on the development of lipid disorders. Amongst the night workers studied, the effects on triglyceridaemic disorders were mainly observed after five to 10 years of exposure, while the consequences for Non-HDL-C and low-HDL-C appeared more gradually, with the excess risk rising by 1–2% per year.

Regarding glycaemic disorders, our results agreed with findings reporting a 10% excess risk of diabetes in male and female shift workers and an estimated increase by 5–7% for every five years of exposure among female shift workers [5, 6]. In our study, only male permanent night workers and female former night workers presented significant higher risks, with an increase of between 2 and 3% per year.

### Strengths and limitations

That the CONSTANCES cohort includes a large number of participants who have undergone a comprehensive medical examination using standardised procedures is a major strength of this study. Indeed, its exploration of different types of night work and exposure duration along individual subjects’ working lives constitutes another of this study’s major strengths. The means of measuring night working types may, however, have led to a wider range of possible types. For instance, while in the present study a night-shift worker was defined as having worked over 50 nights per year, the number of nights worked per month and the range per year could vary greatly. Lastly, the cross-sectional design and the measurement of night work exposure may have led to a potential memorisation bias, thus limiting the causal interpretation of our results.

Future studies would do well to take into account the breaks in participants’ careers when measuring the cumulative exposure duration, as these breaks may have an impact on cardiovascular risks. Moreover, while homogeneous exposure profiles have been identified for permanent and rotating night workers, identifying homogeneous exposure profiles among former night workers is a more complex undertaking. Indeed, both the night work pattern and the time elapsed since ceasing to work at night must be considered when evaluating the potential reversible cardiovascular risk effect.

### Implication for prevention policies in occupational health

All CVRFs were distributed differently according to sex, type of night work and, in some cases, early after the beginning of exposure. For both sexes, these effects persisted even once exposure had ceased. Our results show that night workers need to be monitored for CVRFs throughout their careers by practitioners who specialise in occupational health, even after they have stopped working at night. Although the ideal frequency of such monitoring is not yet clear, existing regular medical visits could offer the opportunity to implement systematic monitoring of these cardiovascular risk factors. Through observing excess risks over certain exposure durations, we can also see that the best management of night-shift workers may even involve a transition to daytime work, facilitated by the occupational health teams in the workplace.

## Conclusions

This study of a large sample of workers demonstrates that the effects of night work on CVRFs varied according to the different types and exposure durations of night work and according to the sex of the participants. Some effects were observed after only five years of exposure. Our findings recommend that early and appropriate monitoring of CVRFs among current and former night-shift workers is needed.

## Supplementary Information

Supplementary Material 1.

## Data Availability

Anyone requesting the data for research purposes can access the deidentified participant data with investigator support. Information on how to access data from the Constances cohort is available online (https://www.constances.fr/en/scientific-area/access-to-constances/). Supporting Documents: 10.13143/inserm_constances.
