# Supplementary material for: Duration of exposure to night work and cardiovascular risk factors: results from 52,234 workers of the CONSTANCES study
Source: BMC Public Health. 2025 Jan 28;25:356. doi: 10.1186/s12889-025-21511-2 (PMC11773728; doi:10.1186/s12889-025-21511-2)
Supplement: Supplementary file 1 — Supplementary Material 1. [file 12889_2025_21511_MOESM1_ESM.pdf]

eFigure 1. Flowchart describing the inclusion of study participants.

Participants in the CONSTANCES cohort:
- **in active employment during recruitment period (2012-2018)**
- **who had worked nights or only days during their working life**

N=62,429

Exclusion of participants due to:
- **history of personal cardio-vascular diseases n=727**
- **history of type 1 diabetes n=118**

Exclusion of participants due to missing values for:
- **cardiovascular risk factors n=4,236**
  - BMI n=1,281
  - Waist circumference n=206
  - Blood pressure n=253
  - Triglyceridaemia n=674
  - Low-HDL-C/Non-HDL-C n=1,814
  - Type 2 diabetes n=8
- **main adjustment factors n=5,114**

Participants included in the analyses n=52,234
- Men n=24,892 (47.7%)
- Women n=27,342 (52.3%)

eTable 1. Socio-demographic, behavioural and occupational characteristics of male workers according to working schedules (day work and types of night work) (n=24,892)

	Day work		Permanent night		Rotating night		Former night	
	n	% or mean (range)	n	% or mean (range)	n	% or mean (range)	n	% or mean (range)
	21,957	88.2	415	1.7	615	2.5	1,905	7.6
**Age**	21,957	44.1 (18.5-71.5)	415	42.5 (20.5-62.5)	615	42.3 (20.0-68.5)	1,905	47.0 (20.5-70.5)
**Educational level**								
< High school degree	4,206	19.2	169	40.7	218	35.5	648	34.0
High school-Bachelor degree	8,064	36.7	183	44.1	352	57.2	907	47.6
≥ Master university degree	9,687	44.1	63	15.2	45	7.3	350	18.4
**Smoking status**								
Current and former smokers	12,165	55.4	265	63.9	391	63.6	1,245	65.4
**Alcohol habits**								
Abstinence	501	2.3	17	4.1	15	2.4	47	2.5
No abusive consumption	7,630	34.7	153	36.9	246	40.0	714	37.5
Risk of abusive consumption	13,826	63.0	245	59.0	354	57.6	1,144	60.0
**No regular leisure time physical activity**	634	2.9	21	5.1	14	2.3	72	3.8
**Score2 (10-year risk of cardiovascular disease)**								
Low	6,787	31.2	111	27.1	147	24.3	415	22.1
Moderate	13,230	60.8	272	66.3	426	70.6	1,271	67.7
High	1,750	8.0	27	6.6	31	5.1	191	10.2
**Total duration of employment (years)**	21,957	21.0 (0.3-54.5)	415	19.8 (0.5-44.0)	615	20.7 (0.5-49.0)	1,905	25.2 (1.0-55.0)
**Jobs (n)**	21,957	3.3 (1.0-27.0)	415	3.4 (1.0-14.0)	615	3.2 (1.0-14.0)	1,905	4.2 (1.0-15.0)
**Work disruptions (n)**	21,957	0.3 (0.0-3.0)	415	0.5 (0.0-3.0)	615	0.4 (0.0-3.0)	1,905	0.6 (0.0-3.0)
**Cumulative duration of night work (permanent or rotating) (years)**								
0	21,957	100.0	0	0.0	0	0.0	0	0.0
]0-5]	-	-	136	32.8	140	22.8	1,064	55.9
]5-10]	-	-	85	20.5	113	18.4	390	20.5
]10-15]	-	-	62	14.9	114	18.5	180	9.4
> 15	-	-	132	31.8	248	40.3	271	14.2
**Permanent night work cumulative duration / cumulative duration of night work (%)**	-	-	415	95.2 (8.2-100.0)	615	2.5 (0.0-96.0)	1,905	44.8 (0.0-100.0)
**Rotating night work cumulative duration / cumulative duration of night work (%)**	-	-	415	4.8 (0.0-91.3)	615	97.5 (4.0-100.0)	1,905	55.2 (0.0-100.0)

Comparison of the frequencies and means between the different working schedules: p values < 0.05 for each of the characteristics.

eTable 2. Socio-demographic, behavioural and occupational characteristics of female workers according to working schedules (day work and types of night work) (n=27,342)

	Day work		Permanent night		Rotating night		Former night	
	n	% or mean (range)	n	% or mean (range)	n	% or mean (range)	n	% or mean (range)
	25,705	94.0	270	1.0	300	1.1	1,067	3.9
**Age**	25,705	44.0 (18.5-71.0)	270	40.8 (21.0-64.5)	300	38.3 (21.5-67.5)	1,067	46.4 (20.0-68.5)
**Educational level**								
< High school degree	3,764	14.6	54	20.0	53	17.7	173	16.2
High school-Bachelor degree	11,771	45.8	145	53.7	170	56.7	573	53.7
≥ Master university degree	10,170	39.6	71	26.3	77	25.6	321	30.1
**Smoking status**								
Current and former smokers	13,200	51.4	145	53.7	158	52.7	615	57.6
**Alcohol habits**								
Abstinence	1,074	4.2	8	3.0	9	3.0	35	3.3
No abusive consumption	10,546	41.0	113	41.8	133	44.3	453	42.5
Risk of abusive consumption	14,085	54.8	149	55.2	158	52.7	579	56.2
**No regular leisure time physical activity**	557	2.2	7	2.6	2	0.7	25	2.3
**Menopausal status**	6,800	26.5	46	17.0	36	12.0	346	32.4
**Substitutive oral hormonal treatment**	950	3.7	8	3.0	8	2.7	50	4.7
**Score2 (10-year risk of cardiovascular disease)**								
Low	20,900	82.0	227	84.4	259	87.5	794	75.1
Moderate	4,494	17.7	42	15.6	37	12.5	258	24.4
High	89	0.3	0	0.0	0	0.0	5	0.5
**Total duration of employment (years)**	25,705	19.9 (0.3-55.0)	270	17.7 (0.5-44.5)	300	15.1 (0.5-49.0)	1,067	22.5 (1.0-49.0)
**Jobs (n)**	25,705	3.3 (1.0-28.0)	270	3.2 (1.0-14.0)	300	2.8 (1.0-7.0)	1,067	4.2 (1.0-21.0)
**Work disruptions (n)**	25,705	0.5 (0.0-3.0)	270	0.5 (0.0-3.0)	300	0.5 (0.0-3.0)	1,067	0.8 (0.0-3.0)
**Cumulative duration of night work (permanent or rotating) (years)**								
0	25,705	100.0	0	0.0	0	0.0	0	0.0
]0-5]	-	-	106	39.3	117	39.0	648	60.7
]5-10]	-	-	59	21.8	76	25.3	225	21.1
]10-15]	-	-	40	14.8	42	14.0	101	9.5
> 15	-	-	65	24.1	65	21.7	93	8.7
**Permanent night work cumulative duration / cumulative duration of night work (%)**	-	-	270	97.0 (11.1-100.0)	300	1.5 (0.0-88.9)	1,067	50.1 (0.0-100.0)
**Rotating night work cumulative duration / cumulative duration of night work (%)**	-	-	270	3.0 (0.0-88.9)	300	98.5 (11.1-100.0)	1,067	49.9 (0.0-100.0)

Comparison of the frequencies and means between the different working schedules: p-value ≤ 0.05 for each of the characteristics, except for alcohol habits, regular leisure time physical activity and substitutive oral hormonal treatment.

eTable 3. Mean and standard deviation (SD) of the cardiovascular risk factors in men and women according to working schedules

	Total sample		Day work		Permanent night		Rotating night		Former night	
**MEN**	Mean	SD	Mean	SD	Mean	SD	Mean	SD	Mean	SD
**BMI (kg/m²)**	25.3	3.7	25.1	3.7	26.1	4.3	26.2	4.0	26.0	4.0
**Waist circumference (cm)**	89.1	10.8	88.8	10.6	90.7	12.1	90.8	11.1	91.4	11.4
**Systolic blood pressure (mmHg)**	131.0	13.5	130.9	13.5	130.6	14.2	131.8	13.5	132.3	13.6
**Diastolic blood pressure (mmHg)**	77.7	9.7	77.5	9.8	77.3	10.0	79.2	9.5	79.0	9.6
**Triglyceridaemia (mmol/L)**	1.2	0.8	1.2	0.8	1.3	0.9	1.5	1.1	1.3	0.9
**HDL-C (mmol/L)**	1.4	0.3	1.4	0.3	1.4	0.3	1.3	0.3	1.4	0.3
**Non-HDL-C (mmol/L)**	4.0	1.0	4.0	1.0	4.0	1.1	4.2	1.1	4.2	1.0
**Glycaemia (mmol/L)**	5.4	0.7	5.4	0.7	5.5	0.8	5.5	0.6	5.5	0.8
**WOMEN**										
**BMI (kg/m²)**	24.0	4.5	23.9	4.5	24.8	5.4	24.1	4.5	24.7	5.1
**Waist circumference (cm)**	78.6	11.3	78.5	11.2	79.5	12.9	78.3	11.4	80.6	12.1
**Systolic blood pressure (mmHg)**	119.9	14.4	119.9	14.4	120.2	13.7	118.9	13.2	121.5	15.8
**Diastolic blood pressure (mmHg)**	73.4	9.3	73.4	9.3	73.6	9.1	72.6	9.3	74.1	9.5
**Triglyceridaemia (mmol/L)**	0.9	0.5	0.9	0.5	1.0	0.6	1.0	0.5	1.0	0.5
**HDL-C (mmol/L)**	1.7	0.4	1.7	0.4	1.6	0.4	1.6	0.4	1.7	0.4
**Non-HDL-C (mmol/L)**	3.7	1.0	3.7	1.0	3.6	0.9	3.6	1.0	3.8	1.0
**Glycaemia (mmol/L)**	5.1	0.6	5.1	0.6	5.1	0.6	5.0	0.4	5.2	0.9

BMI: body mass index; HDL: high-density lipoprotein

eTable 4. Frequency of clinical and biological cardiovascular risk factors according to working schedules and health behavioural characteristics of male workers (n=24,892)

	Obesity		Central obesity		High arterial blood pressure		High triglyceridaemia		Low-HDL-C		Non-HDL-C		Type 2 diabetes	
	n	%	n	%	n	%	n	%	n	%	n	%	n	%
	2,548	10.2	3,026	12.2	7,725	31.0	4,494	18.1	4,625	18.6	11,137	44.7	723	2.9
**Working schedules**														
Day work	2,105	9.6	2,523	11.5	6,721	30.6	3,833	17.5	3,977	18.1	9,628	43.9	603	2.8
Permanent night	65	15.7	69	16.6	119	28.7	84	20.2	81	19.5	184	44.3	20	4.8
Rotating night	102	16.6	100	16.3	208	33.8	169	27.5	133	21.6	307	49.9	20	3.3
Former night	276	14.5	334	17.5	677	35.5	408	21.4	434	22.8	1,018	53.4	80	4.2
**Smoking status**														
Never	902	8.3	1,035	9.6	3,084	28.5	1,547	14.3	1,721	15.9	4,301	39.7	219	2.0
Current and former	1,646	11.7	1,991	14.2	4,641	33.0	2,947	21.0	2,904	20.7	6,836	48.6	504	3.6
**Alcohol habits**														
Abstinence	82	14.1	87	15.0	170	29.3†	120	20.7†	133	22.9	239	41.2	33	5.7
No abusive consumption	946	10.8	1,117	12.8	2,744	31.4	1,566	17.9	1,820	20.8	4,019	46.0	267	3.1
Risk of abusive consumption	1,520	9.8	1,822	11.7	4,811	30.9	2,808	18.0	2,672	17.2	6,879	44.2	423	2.7
**Regular leisure time physical activity**														
Yes	2,375	9.8	2,847	11.8	7,434	30.8	4,270	17.7	4,417	18.3	10,732	44.4	678	2.8
No	173	23.4	179	24.2	291	39.3	224	30.2	208	28.1	405	54.7	45	6.1

Obesity: BMI ≥ 30 kg/m²; Central obesity: WC ≥102 cm; HBP: 140/90 mmHg or medical history or treatment; HighTG: ≥1.7 mmol/L or treatment; Low-HDL-C: <1.04 mmol/L or treatment; Non-HDL-C: ≥4.2 mmol/L or treatment; Type 2 diabetes: glycaemia ≥7 mmol/L or type 2 diabetes history or treatment.

The frequency of each clinical and biological cardiovascular risk factor was statistically significantly different at the 5% level across categories of working schedules and health behavioural characteristics, except for those marked †.

eTable 5. Frequency of clinical and biological cardiovascular risk factors according to working schedules and health behavioural characteristics of female workers (n=27,342)

	Obesity		Central obesity		High arterial blood pressure		High triglyceridaemia		Low-HDL-C		Non-HDL-C		Type 2 diabetes	
	n	%	n	%	n	%	n	%	n	%	n	%	n	%
	2,702	9.9	5,222	19.1	4,472	16.4	1,815	6.6	4,971	18.2	8,031	29.4	445	1.6
**Working schedules**														
Day work	2,489	9.7	4,839	18.8	4,155	16.2	1,670	6.5	4,613	18.0	7,534	29.3	405	1.6
Permanent night	37	13.7	60	22.2	42	15.6	30	11.1	66	24.4	66	24.4	6	2.2
Rotating night	34	11.3	57	19.0	44	14.7	25	8.3	60	20.0	79	26.3	4	1.3
Former night	142	13.3	266	24.9	231	21.7	90	8.4	232	21.7	352	33.0	30	2.8
**Smoking status**														
Never	1,361	10.3	2,491	18.8†	2,223	16.8	797	6.0	2,291	17.3	3,807	28.8	225	1.7†
Current and former	1,341	9.5	2,731	19.3	2,249	15.9	1,018	7.2	2,680	19.0	4,224	29.9	220	1.6
**Alcohol habits**														
Abstinence	213	18.9	330	29.3	227	20.2	111	9.9	322	28.6	371	32.9	46	4.1
No abusive consumption	1,325	11.8	2,344	20.8	1,975	17.6	790	7.0	2,342	20.8	3,590	31.9	233	2.1
Risk of abusive consumption	1,164	7.8	2,548	17.0	2,270	15.2	914	6.1	2,307	15.4	4,070	27.2	166	1.1
**Regular leisure time physical activity**														
Yes	2,586	9.7	5,041	18.8	4,350	16.3	1,745	6.5	4,826	18.0	7,837	29.3†	427	1.6
No	116	19.6	181	30.6	122	20.6	70	11.8	145	24.5	194	32.8	18	3.1

Obesity: BMI ≥ 30 kg/m²; Central obesity: WC ≥88 cm; HBP: 140/90 mmHg or medical history or treatment; HighTG: ≥1.7 mmol/L or treatment; Low-HDL-C: <1.29 mmol/L or treatment; Non-HDL-C: ≥4.2 mmol/L or treatment; Type 2 diabetes: glycaemia ≥7 mmol/L or type 2 diabetes history or treatment.
The frequency of each clinical and biological cardiovascular risk factor was statistically significantly different at the 5% level across categories of working schedules and health behavioural characteristics, except for those marked †.

eTable 6. Socio-demographic and occupational characteristics of the participants included in and excluded from the study, for all workers

	Included participants		Excluded participants due to missing values for cardiovascular risk factors		Excluded participants due to missing values for adjustment factors	
	n	% or mean (range)	n	% or mean (range)	n	% or mean (range)
	52,234	84.8	4,236	6.9	5,114	8.3
**Sex**						
Women	27,342	52.3	2,326	54.9	2,997	58.6
Men	24,892	47.7	1,910	45.1	2,117	41.4
**Age**	52,234	44.1 (18.5-71.5)	4,236	41.4 (18.5-70.5)	5,114	47.4 (18.5-71.5)
**Educational level**[a]						
< High school degree	9,285	17.8	725	17.8	1,794	41.1
High school-Bachelor degree	22,165	42.4	1,831	45.0	1,641	37.6
≥ Master university degree	20,784	39.8	1,513	37.2	929	21.3
**Working schedules**						
Day work	47,662	91.2	3,832	90.5	4,652	91.0†
Permanent night	685	1.3	69	1.6	75	1.5
Rotating night	915	1.8	118	2.8	103	2.0
Former night	2,972	5.7	217	5.1	284	5.6
**Total duration of employment (years)**	52,234	20.5 (0.3-55.0)	4,236	17.9 (0.5-54.0)	5,114	23.1 (0.3-56.0)

[a] Education level: 1.5% missing values for participants excluded due to missing values for cardiovascular risk factors and 16% missing values for participants excluded due to missing values for adjustment factors.

The frequency of socio-demographic and occupational characteristics was statistically significantly different at the 5% level across included and excluded participants, except for this marked †.

eTable 7. Association between different types of night work, adjustment factors and cardiovascular risk factors among male and female workers.

		Men (n=24,892)	Women (n=27,342)
		**OR (95%CI)**	**OR (95%CI)**
**BMI≥30 kg/m²**			
**Work schedule [a]**	Permanent night	1.50 (1.14-1.97)**	1.42 (0.98-2.03)*
	Rotating night	1.66 (1.32-2.07)**	1.20 (0.84-1.73)
	Former night	1.24 (1.08-1.42)**	1.34 (1.11-1.61)**
**Age**		1.03 (1.03-1.04)**	1.02 (1.01-1.02)**
**Educational level [b]**	High school-Bachelor degree	1.58 (1.42-1.75)**	1.77 (1.61-1.96)**
	< High school degree	2.21 (1.98-2.47)**	2.69 (2.39-3.04)**
**Smoking status [c]**	Current and former smokers	1.22 (1.11-1.34)**	1.00 (0.92-1.08)
**No regular leisure time physical activity**		2.71 (2.26-3.24)**	2.25 (1.82-2.78)**
**Alcohol habits**		0.99 (0.97-1.01)	0.86 (0.84-0.88)**
**Menopausal status**		-	0.96 (0.85-1.09)
**Substitutive oral hormonal treatment**		-	0.56 (0.43-0.72)**
**Waist circumference ≥102 ♂ / 88 ♀ cm**			
**Work schedule [a]**	Permanent night	1.42 (1.08-1.87)**	1.28 (0.95-1.72)*
	Rotating night	1.43 (1.14-1.80)**	1.14 (0.85-1.54)
	Former night	1.25 (1.10-1.43)**	1.30 (1.12-1.51)**
**Age**		1.06 (1.05-1.06)**	1.03 (1.03-1.04)**
**Educational level [b]**	High school-Bachelor degree	1.45 (1.31-1.59)**	1.50 (1.40-1.62)**
	< High school degree	2.05 (1.84-2.27)**	2.17 (1.98-2.38)**
**Smoking status [c]**	Current and former smokers	1.26 (1.16-1.37)**	1.05 (0.98-1.12)
**No regular leisure time physical activity**		2.39 (2.00-2.87)**	1.94 (1.62-2.33)**
**Alcohol habits**		1.00 (0.99-1.02)	0.93 (0.92-0.95)**
**Menopausal status**		-	1.02 (0.93-1.12)
**Substitutive oral hormonal treatment**		-	0.66 (0.56-0.78)**
**High arterial blood pressure (SBP / DBP≥140 / 90 mm Hg or hypertension history or treatment)**			
**Work schedule [a]**	Permanent night	0.89 (0.71-1.11)	1.24 (0.87-1.76)
	Rotating night	1.18 (0.98-1.41)*	1.40 (0.99-1.98)*
	Former night	0.98 (0.88-1.09)	1.24 (1.06-1.45)**
**Age**		1.06 (1.06-1.07)**	1.09 (1.09-1.10)**
**Educational level [b]**	High school-Bachelor degree	1.34 (1.25-1.43)**	1.42 (1.31-1.54)**
	< High school degree	1.68 (1.56-1.81)**	1.88 (1.71-2.08)**
**Smoking status [c]**	Current and former smokers	0.96 (0.91-1.02)	0.87 (0.81-0.94)**
**No regular leisure time physical activity**		1.49 (1.27-1.75)**	1.46 (1.17-1.81)**
**Alcohol habits**		1.05 (1.03-1.06)**	0.99 (0.97-1.01)
**Menopausal status**		-	0.92 (0.84-1.02)
**Substitutive oral hormonal treatment**		-	0.92 (0.79-1.07)
**High triglyceridaemia (≥1.7 mmol/L or treatment)**			
**Work schedule [a]**	Permanent night	1.11 (0.86-1.41)	1.88 (1.28-2.77)**
	Rotating night	1.71 (1.42-2.05)**	1.48 (0.98-2.25)*
	Former night	1.10 (0.98-1.24)	1.21 (0.97-1.51)*
**Age**		1.02 (1.02-1.03)**	1.02 (1.01-1.03)**
**Educational level [b]**	High school-Bachelor degree	1.20 (1.11-1.30)**	1.44 (1.28-1.62)**
	< High school degree	1.38 (1.26-1.51)**	1.99 (1.73-2.29)**
**Smoking status [c]**	Current and former smokers	1.42 (1.32-1.52)**	1.19 (1.08-1.32)**
**No regular leisure time physical activity**		1.97 (1.67-2.32)**	1.93 (1.49-2.50)**

**Alcohol habits**		1.01 (1.00-1.03)	0.97 (0.95-1.00)*
**Menopausal status**		-	1.38 (1.20-1.60)**
**Substitutive oral hormonal treatment**		-	0.80 (0.63-1.02)*
**Low-HDL-C (<1.04 ♂ / 1.29 ♀ mmol/L or treatment)**			
**Work schedule** [a]	Permanent night	1.02 (0.80-1.31)	1.36 (1.03-1.81)**
	Rotating night	1.19 (0.98-1.45)*	1.02 (0.77-1.36)
	Former night	1.15 (1.02-1.29)**	1.24 (1.07-1.45)**
**Age**		1.02 (1.02-1.03)**	0.99 (0.99-0.99)**
**Educational level** [b]	High school-Bachelor degree	1.10 (1.02-1.19)**	1.30 (1.21-1.39)**
	< High school degree	1.30 (1.19-1.42)**	1.73 (1.57-1.90)**
**Smoking status** [c]	Current and former smokers	1.37 (1.28-1.47)**	1.27 (1.19-1.36)**
**No regular leisure time physical activity**		1.67 (1.42-1.97)**	1.41 (1.17-1.71)**
**Alcohol habits**		0.92 (0.91-0.94)**	0.86 (0.85-0.88)**
**Menopausal status**		-	1.03 (0.93-1.14)
**Substitutive oral hormonal treatment**		-	0.88 (0.74-1.06)
**Non-HDL-C (≥4.2 mmol/L or treatment)**			
**Work schedule** [a]	Permanent night	1.02 (0.83-1.25)	0.94 (0.70-1.27)
	Rotating night	1.31 (1.11-1.55)**	1.28 (0.97-1.69)*
	Former night	1.18 (1.07-1.30)**	1.00 (0.87-1.15)
**Age**		1.06 (1.06-1.06) **	1.06 (1.06-1.07)**
**Educational level** [b]	High school-Bachelor degree	1.24 (1.17-1.32)**	1.34 (1.26-1.43)**
	< High school degree	1.33 (1.24-1.44)**	1.64 (1.51-1.79)**
**Smoking status** [c]	Current and former smokers	1.23 (1.16-1.30)**	1.04 (0.98-1.10)
**No regular leisure time physical activity**		1.52 (1.31-1.78)**	1.25 (1.03-1.51)**
**Alcohol habits**		1.00 (0.99-1.02)	0.94 (0.92-0.95)**
**Menopausal status**		-	1.67 (1.54-1.82)**
**Substitutive oral hormonal treatment**		-	0.73 (0.64-0.83)**
**Type 2 diabetes (glycaemia ≥7 mmol/L or type 2 diabetes history or treatment)**			
**Work schedule** [a]	Permanent night	1.86 (1.16-2.98)**	1.63 (0.71-3.70)
	Rotating night	1.27 (0.80-2.03)	1.09 (0.40-2.97)
	Former night	1.13 (0.88-1.44)	1.64 (1.12-2.40)**
**Age**		1.10 (1.09-1.11)**	1.06 (1.04-1.07)**
**Educational level** [b]	High school-Bachelor degree	1.41 (1.15-1.71)**	1.32 (1.04-1.67)**
	< High school degree	2.15 (1.76-2.61)**	1.88 (1.44-2.46)**
**Smoking status** [c]	Current and former smokers	1.41 (1.19-1.66)**	1.02 (0.84-1.23)
**No regular leisure time physical activity**		2.22 (1.61-3.06)**	1.94 (1.19-3.14)**
**Alcohol habits**		0.96 (0.93-1.00)**	0.84 (0.79-0.89)**
**Menopausal status**		-	0.89 (0.68-1.16)
**Substitutive oral hormonal treatment**		-	0.33 (0.17-0.65)**

BMI: body mass index; SBP: systolic blood pressure; DBP: diastolic blood pressure; HDL: high-density lipoprotein.
[a] reference group: Day work; [b] reference group: ≥ Master university degree; [c] reference group: Non smokers
**: $p \leq 0.05$; * $p < 0.10$.

eTable 8. Association between night work exposure and 10-year risk of cardiovascular disease (SCORE2), for all workers

	Moderate-high SCORE2			
	OR	95%CI		
**Profile of work pattern**				
Day work	1			
Permanent night	**1.43**	**1.23**	**-**	**1.66**
Rotating night	**1.72**	**1.51**	**-**	**1.97**
Former night	**2.01**	**1.87**	**-**	**2.18**
**Cumulative night work duration (years)**				
**Among permanent night workers**				
Test smooth trend vs. linear trend	*p=0.771*			
0	1			
5	1.12	0.89	-	1.39
10	1.28	1.00	-	1.64*
15	**1.53**	**1.16**	**-**	**2.02**
20	**1.86**	**1.34**	**-**	**2.57**
Linear trend (per year)	***1.03***	***1.02***	**-**	***1.04***
**Among rotating night workers**				
Test smooth trend vs. linear trend	*p=0.065*			
0	1			
5	1.07	0.88	-	1.30
10	**1.48**	**1.19**	**-**	**1.83**
15	**2.13**	**1.69**	**-**	**2.68**
20	**2.90**	**2.21**	**-**	**3.82**
Linear trend (per year)	***1.05***	***1.04***	**-**	***1.06***
**Among former night workers**				
Test smooth trend vs. linear trend	*p<0.001*			
0	1			
5	**1.82**	**1.64**	**-**	**2.02**
10	**2.39**	**2.07**	**-**	**2.74**
15	**2.84**	**2.36**	**-**	**3.41**
20	**3.38**	**2.68**	**-**	**4.27**
Linear trend (per year)	***1.08***	***1.07***	**-**	***1.09***

In bold: p ≤ 0.05; * p < 0.10.

eTable 9. Association between cumulative night work duration at time period (years) and cardiovascular risk factors among permanent night workers compared with day workers

	Men (n=22,372)				Women (n=25,975)			
**Cumulative night work duration, years**	**OR**	**95%CI**			**OR**	**95%CI**		
**BMI ≥30 kg/m²**								
Test smooth trend vs. linear trend	***p=0.003***				*p=0.841*			
0	1				1			
5	**1.76**	**1.17**	**-**	**2.62**	1.30	0.75		2.28
10	**2.08**	**1.36**	**-**	**3.18**	1.27	0.70		2.33
15	**1.78**	**1.12**	**-**	**2.82**	1.22	0.62		2.40
20	1.32	0.76	-	2.29	1.46	0.64		3.32
Linear trend (per year)	*1.01*	*0.99*	-	*1.03*	*1.02*	*1.00*	-	1.05*
**Waist circumference ≥102 ♂ / ≥88 ♀ cm**								
Test smooth trend vs. linear trend	***p=0.028***				*p=0.747*			
0	1				1			
5	**1.64**	**1.09**	**-**	**2.46**	1.18	0.75	-	1.87
10	**1.90**	**1.24**	**-**	**2.92**	1.25	0.77	-	2.04
15	1.58	1.00	-	2.50*	1.34	0.79	-	2.29
20	1.14	0.66	-	1.95	1.59	0.83	-	3.03
Linear trend (per year)	*1.01*	*0.99*	-	*1.03*	***1.02***	***1.00***	**-**	***1.04***
**High arterial blood pressure (SBP / DBP≥140 / 90 mm Hg or hypertension history or treatment)**								
Test smooth trend vs. linear trend	*p=0.730*				*p=0.800*			
0	1				1			
5	1.06	0.76	-	1.48	1.28	0.73	-	2.24
10	1.03	0.71	-	1.48	1.29	0.73	-	2.30
15	0.86	0.58	-	1.28	1.18	0.63	-	2.18
20	0.75	0.48	-	1.17	1.33	0.64	-	2.77
Linear trend (per year)	*0.99*	*0.98*	-	*1.00*	*1.01*	*0.99*	-	*1.04*
**High triglyceridaemia (≥1.7 mmol/L or treatment)**								
Test smooth trend vs. linear trend	*p=0.099*				*p=0.402*			
0	1				1			
5	1.36	0.95	-	1.95*	1.72	0.94	-	3.17*
10	1.18	0.79	-	1.76	**2.23**	**1.19**	**-**	**4.18**
15	0.83	0.53	-	1.30	1.83	0.89	-	3.77*
20	0.75	0.45	-	1.26	1.61	0.65	-	3.98
Linear trend (per year)	*0.99*	*0.98*	-	*1.01*	***1.04***	***1.01***	**-**	***1.06***
**Low-HDL-C (<1.04 ♂ / <1.29 ♀ mmol/L or treatment)**								
Test smooth trend vs. linear trend	*p=0.516*				*p=0.545*			
0	1				1			
5	1.12	0.78	-	1.62	1.27	0.83	-	1.95
10	1.18	0.79	-	1.76	1.47	0.92	-	2.35
15	1.07	0.69	-	1.66	1.28	0.74	-	2.22
20	0.93	0.56	-	1.54	1.12	0.55	-	2.28
Linear trend (per year)	*1.00*	*0.98*	-	*1.01*	*1.02*	*1.00*	-	1.04*
**Non-HDL-C (≥4.2 mmol/L or treatment)**								
Test smooth trend vs. linear trend	*p=0.544*				*p=0.087*			
0	1				1			
5	1.07	0.79	-	1.46	1.09	0.69		1.72
10	0.95	0.68	-	1.33	0.84	0.51		1.39
15	0.88	0.62	-	1.26	0.59	0.34		1.05
20	0.82	0.55	-	1.23	0.80	0.41		1.56
Linear trend (per year)	*0.99*	*0.98*	-	*1.01*	*1.00*	*0.98*	-	*1.02*
**Type 2 diabetes (glycaemia ≥7 mmol/L or type 2 diabetes history or treatment)**								
Test smooth trend vs. linear trend	*p=0.243*				*p=0.580*			
0	1				1			
5	1.66	0.79	-	3.51	0.95	0.26	-	3.42
10	**2.19**	**1.01**	**-**	**4.73**	0.96	0.23	-	4.03
15	**2.34**	**1.08**	**-**	**5.07**	1.15	0.25	-	5.21
20	1.89	0.79	-	4.54	2.00	0.42	-	9.52
Linear trend (per year)	***1.02***	***1.00***	**-**	***1.05***	***1.04***	***1.00***	**-**	***1.09***

Generalised additive model with smooth functions of age and night work duration and adjusted for age, educational level, smoking status, alcohol habits and regular leisure time physical activity, along with menopausal status and substitutive oral hormonal treatment in female models.
BMI: body mass index; SBP: systolic blood pressure; DBP: diastolic blood pressure; HDL: high-density lipoprotein. In bold: p ≤ 0.05; * p < 0.10.

eTable 10. Association between cumulative night work duration at time period (years) and cardiovascular risk factors among rotating night workers compared with day workers

	Men (n=22,572)		Women (n=26,005)	
Cumulative night work duration, years	OR	95%CI	OR	95%CI
**BMI ≥30 kg/m²**				
Test smooth trend vs. linear trend	*p=0.152*		*p=0.592*	
0	1		1	
5	**1.46**	**1.03 - 2.08**	1.28	0.78 - 2.08
10	**1.66**	**1.15 - 2.38**	1.42	0.82 - 2.47
15	**1.63**	**1.14 - 2.32**	1.28	0.65 - 2.49
20	**1.71**	**1.16 - 2.51**	1.07	0.46 - 2.48
Linear trend (per year)	***1.02***	***1.01 - 1.03***	*1.01*	*0.98 - 1.03*
**Waist circumference ≥102 ♂ / ≥88 ♀ cm**				
Test smooth trend vs. linear trend	*p=0.095*		*p=0.193*	
0	1		1	
5	**1.49**	**1.05 - 2.14**	1.25	0.84 - 1.86
10	**1.60**	**1.11 - 2.31**	1.26	0.79 - 2.00
15	**1.45**	**1.01 - 2.08**	1.06	0.61 - 1.84
20	1.33	0.89 - 1.98	0.86	0.43 - 1.71
Linear trend (per year)	***1.01***	***1.00 - 1.02***	*1.01*	*0.99 - 1.03*
**High arterial blood pressure (SBP / DBP ≥140 / 90 mm Hg or hypertension history or treatment)**				
Test smooth trend vs. linear trend	*p=0.216*		*p=0.105*	
0	1		1	
5	1.29	0.99 - 1.70*	**1.73**	**1.08 - 2.80**
10	1.28	0.97 - 1.71*	1.67	0.97 - 2.85*
15	1.13	0.85 - 1.51	1.32	0.71 - 2.43
20	1.04	0.76 - 1.44	1.15	0.55 - 2.41
Linear trend (per year)	*1.00*	*0.99 - 1.01*	*1.01*	*0.99 - 1.03*
**High triglyceridaemia (≥1.7 mmol/L or treatment)**				
Test smooth trend vs. linear trend	***p=0.011***		*p=0.441*	
0	1		1	
5	**1.52**	**1.15 - 2.02**	1.38	0.77 - 2.45
10	**1.76**	**1.31 - 2.35**	1.57	0.81 - 3.03
15	**1.72**	**1.28 - 2.29**	1.65	0.77 - 3.56
20	**1.48**	**1.06 - 2.06**	1.35	0.52 - 3.50
Linear trend (per year)	***1.02***	***1.01 - 1.03***	*1.02*	*1.00 - 1.05**
**Low HDL (<1.04 ♂ / <1.29 ♀ mmol/L or treatment)**				
Test smooth trend vs. linear trend	*p=0.853*		*p=0.544*	
0	1		1	
5	1.07	0.79 - 1.46	0.99	0.67 - 1.45
10	1.16	0.84 - 1.61	0.99	0.63 - 1.56
15	1.26	0.92 - 1.73	1.07	0.61 - 1.86
20	1.24	0.87 - 1.77	1.06	0.53 - 2.14
Linear trend (per year)	*1.01*	*1.00 - 1.02**	*1.00*	*0.98 - 1.02*
**Non-HDL-C (≥4.2 mmol/L or treatment)**				
Test smooth trend vs. linear trend	*p=0.422*		*p=0.462*	
0	1		1	
5	1.06	0.81 - 1.37	1.14	0.77 - 1.69
10	1.20	0.91 - 1.57	1.15	0.73 - 1.80
15	**1.36**	**1.04 - 1.78**	1.19	0.71 - 1.99
20	1.36	1.00 - 1.84*	1.17	0.62 - 2.18
Linear trend (per year)	***1.01***	***1.00 - 1.02***	*1.02*	*1.00 - 1.04**
**Type 2 diabetes (glycaemia ≥7 mmol/L or type 2 diabetes history or treatment)**				
Test smooth trend vs. linear trend	*p=0.708*		*p=0.505*	
0	1		1	
5	0.94	0.44 - 2.02	1.88	0.62 - 5.67
10	1.00	0.44 - 2.30	1.12	0.29 - 4.27
15	1.30	0.60 - 2.81	0.37	0.08 - 1.75
20	1.45	0.67 - 3.15	0.18	0.03 - 1.01
Linear trend (per year)	*1.01*	*0.99 - 1.03*	*0.95*	*0.90 - 1.01**

Generalised additive model with smooth functions of age and night work duration and adjusted for age, educational level, smoking status, alcohol habits and regular leisure time physical activity, along with menopausal status and substitutive oral hormonal treatment in female models.
BMI: body mass index; SBP: systolic blood pressure; DBP: diastolic blood pressure; HDL: high-density lipoprotein. In bold: p ≤ 0.05; * p < 0.10.

eTable 11. Association between cumulative night work duration at time period (years) and cardiovascular risk factors among former night workers compared with day workers

	Men (n=23,862)				Women (n=26,772)			
**Cumulative night work duration, years**	**OR**	**95%CI**			**OR**	**95%CI**		
**BMI ≥30 kg/m²**								
Test smooth trend vs. linear trend	*p=0.081*				*p=0.007*			
0	1				1			
5	**1.22**	**1.02**	**-**	**1.47**	**1.47**	**1.15**	**-**	**1.89**
10	**1.35**	**1.08**	**-**	**1.70**	1.39	1.00	-	1.93*
15	1.27	0.96	-	1.69*	1.09	0.68	-	1.76
20	1.12	0.80	-	1.59	0.83	0.43	-	1.59
Linear trend (per year)	*1.01*	*1.00*	*-*	*1.02**	*1.01*	*0.99*	*-*	*1.03*
**Waist circumference ≥102 ♂ / ≥88 ♀ cm**								
Test smooth trend vs. linear trend	*p=0.410*				***p=0.0001***			
0	1				1			
5	1.17	0.98	-	1.40*	**1.43**	**1.18**	**-**	**1.75**
10	**1.25**	**1.01**	**-**	**1.55**	1.20	0.92	-	1.56
15	1.21	0.93	-	1.57	0.93	0.64	-	1.35
20	1.15	0.84	-	1.58	0.77	0.47	-	1.26
Linear trend (per year)	***1.01***	***1.00***	***-***	***1.02***	*1.00*	*0.99*	*-*	*1.02*
**High arterial blood pressure (SBP / DBP ≥140 / 90 mm Hg or hypertension history or treatment)**								
Test smooth trend vs. linear trend	*p=0.580*				*p=0.018*			
0	1				1			
5	1.02	0.89	-	1.18	**1.34**	**1.08**	**-**	**1.66**
10	1.05	0.88	-	1.25	**1.41**	**1.07**	**-**	**1.85**
15	0.98	0.79	-	1.23	1.08	0.75	-	1.56
20	0.97	0.75	-	1.27	0.87	0.54	-	1.40
Linear trend (per year)	*1.00*	*0.99*	*-*	*1.01*	*1.01*	*0.99*	*-*	*1.02*
**High triglyceridaemia (≥1.7 mmol/L or treatment)**								
Test smooth trend vs. linear trend	*p=0.121*				*p=0.082*			
0	1				1			
5	1,16	1.00	-	1,36*	1.25	0.92	-	1.70
10	1,10	0,90	-	1,35	1.21	0.81	-	1.81
15	0,99	0,77	-	1,28	0.97	0.55	-	1.71
20	0,92	0,67	-	1,25	0.80	0.38	-	1.69
Linear trend (per year)	*1.00*	*0.99*	*-*	*1.01*	*1.01*	*0.99*	*-*	*1.03*
**Low-HDL-C (<1.04 ♂ / <1.29 ♀ mmol/L or treatment)**								
Test smooth trend vs. linear trend	*p=0.100*				***p=0.001***			
0	1				1			
5	1.13	0.97	-	1.32	**1.35**	**1.10**	**-**	**1.65**
10	1.10	0.90	-	1.34	**1.38**	**1.05**	**-**	**1.81**
15	1.10	0.86	-	1.41	1.07	0.72	-	1.59
20	1.18	0.88	-	1.58	0.72	0.42	-	1.26
Linear trend (per year)	*1.00*	*0.99*	*-*	*1.01*	*1.01*	*0.99*	*-*	*1.03*
**Non-HDL-C (≥4.2 mmol/L or treatment)**								
Test smooth trend vs. linear trend	*p=0.227*				*p=0.849*			
0	1				1			
5	**1.17**	**1.02**	**-**	**1.34**	0.98	0.81	-	1.19
10	1.17	0.98	-	1.39*	0.94	0.74	-	1.21
15	1.12	0.90	-	1.40	0.94	0.68	-	1.31
20	1.18	0.90	-	1.53	0.87	0.56	-	1.33
Linear trend (per year)	***1.01***	***1.00***	***-***	***1.02***	*0.99*	*0.98*	*-*	*1.01*
**Type 2 diabetes (glycaemia ≥7 mmol/L or type 2 diabetes history or treatment)**								
Test smooth trend vs. linear trend	*p=0.395*				*p=0.062*			
0	1				1			
5	1.07	0.76	-	1.49	1.39	0.80	-	2.40
10	0.85	0.55	-	1.31	1.45	0.72	-	2.91
15	0.82	0.49	-	1.36	1.22	0.46	-	3.23
20	1.01	0.58	-	1.77	0.87	0.23	-	3.22
Linear trend (per year)	*1.00*	*1.00*	*-*	*1.00*	***1.03***	***1.00***	***-***	***1.07***

Generalised additive model with smooth functions of age and night work duration and adjusted for age, educational level, smoking status, alcohol habits and regular leisure time physical activity, along with menopausal status and substitutive oral hormonal treatment in female models.
BMI: body mass index; SBP: systolic blood pressure; DBP: diastolic blood pressure; HDL: high-density lipoprotein. In bold: p ≤ 0.05; * p < 0.10.
